# Biomarkers, Inflammation, and Bipolar Disorder: Association Between the Improvement of Bipolar Disorder Severity and the Improvement in C-Reactive Protein Levels After 7 Days of Inpatient Treatment

**DOI:** 10.3389/fpsyt.2021.803034

**Published:** 2021-12-14

**Authors:** Alessandro Cuomo, Despoina Koukouna, Alessandro Spiti, Giovanni Barillà, Arianna Goracci, Simone Bolognesi, Andrea Fagiolini

**Affiliations:** Dipartimento di Medicina Molecolare e dello Sviluppo, Dipartimento di Psichiatria, University of Siena, Siena, Italy

**Keywords:** bipolar disorder, inflammation, CRP, C-reactive protein, biomarker, inpatients

## Abstract

**Introduction:** Compared to the general population, people with severe mental illness (SMI) have a poorer health status and a higher mortality rate, with a 10–20-year reduction in life expectancy. Excess mortality and morbidity in SMI have been explained by intertwined components. Inflammatory processes could increase the morbidity and mortality risk in patients with bipolar disorder (BD) because of a bidirectional interaction between BD and conditions related to inflammation. This pilot study aimed to evaluate the relationship between C-Reactive-Protein (CRP) and bipolar disorder severity.

**Methods:** A retrospective observational study was conducted on 61 hospitalized patients with bipolar disorder. CRP was measured at admission to inpatient treatment (T0) and after seven days from the admission (T1). Clinical Global Impression for Depression, Mania and Overall Bipolar Illness were recorded at T0 and T1. Comparisons among the recorded CRP values were determined through the paired *t*-test. Correlations between CRP and CGI scores were determined through Spearman's correlation coefficient at T0 and T1.

**Results:** A statistically significant decrease in CRP values was observed after 7 days of hospitalization (*p* < 0.001) and positive significant correlations emerged between CRP and CGI scores at T0 and T1.

**Conclusion:** Patients admitted to the inpatient unit reported a statistically significant decrease of CRP values during the first 7 days of treatment. Although the direction of the relationship between BP severity and inflammation status continues to remain unclear, this study showed a relationship between the improvement of bipolar disease symptoms and the improvement of the inflammatory marker CRP.

## Introduction

The morbidity, mortality, and suffering associated with severe mental disorders are not only a result of psychiatric symptoms and their attendant dysfunction (1). Medical disease and medical risk factors are common in patients with mental illness in general and in patients with bipolar disorder (BD) in particular. Physical illness affects the course, severity, and treatment of bipolar disorder (2–6), and lead to even greater morbidity, mortality, and disability (7).

Many physical illnesses have been cited as highly prevalent in patients with bipolar disease, the most common of which are obesity, cardiovascular disease, diabetes, and thyroid disease. Since patients with bipolar disorder spend most of their time in the depressive phase of the illness, there is often a loss of the discipline and motivation required to reduce such physical risk factors.

Katon has established a clear relationship between depression and several negative health behaviors including poor diet, excessive eating, smoking, abuse of substances, and a sedentary lifestyle (8). For example, mortality due to cardiovascular disease has been reported as doubled in patients with BD (9). Moreover, abnormalities in the homeostatic balance between the sympathetic and the parasympathetic systems, with loss of heart rate variability (HRV), sympathoadrenal activation, hypothalamic-pituitary-adrenal (HPA) axis activation, immune system dysregulation resulting in a pro-inflammatory status, platelet activation, and endothelial dysfunction have been observed in many patients with BD, major depressive disorder (MDD), and other psychiatric disorders (10). The study of inflammatory biomarkers and their change in the different phases of BD may contribute to the research about the etiology and treatment of BD (11).

Many studies have identified neuroinflammatory mechanisms of bipolar disorder (BD), showing how these mechanisms may impact the disease progression and the effectiveness of drug treatment.

For instance, a correlation between autoimmune processes and increased expression of psychiatric disorders is supported by the increased risk of patients with systemic autoimmune diseases developing BD (12). Although such chronic immune dysfunction appears to contribute significantly to developing comorbidities in BD, the direction of this cause-effect relationship is still unclear. Specifically, it is not yet clear whether BD increases the risk for immune dysfunction or whether a pre-existing inflammatory condition increases the risk of BD. The most recent hypothesis suggests a bidirectional interaction between BD and conditions related to inflammation and that these reinforce each other; moreover, specific genetic and environmental factors contribute to increasing the risk (13).

Several biologic mechanisms may contribute to the increased mortality risk from natural causes found among patients with bipolar disorder, such as dysregulation of the hypo-thalamic-pituitary-adrenal axis; the dysregulation of the autonomic nervous system may also lead to insulin resistance and may worsen metabolic syndrome (14), and pharmacological treatment.

Measuring neuroinflammation through pro and anti-inflammatory cytokines should determine a positive increase in the treatment of bipolar disorder. Rosenblat et al. hypothesized a simultaneous trend in mood levels, cognitive functions, and inflammatory markers in patients with bipolar disorder (13). According to this scheme, cytokine levels increase chronically and can increase both during depressive and manic episodes. Specifically, Fernandes et al. showed an increase in C reactive protein (CRP) more markedly in the acute phase of mania (15, 16), while Klaus Munkholm et al. showed an altered leukocyte component in patients with bipolar disorder compared to the healthy control group on a sample of 300 blood draws (17).

High levels of CRP have been found in various psychiatric disorders but particularly in schizophrenia, depressive disorders, bipolar disorder (18). Scientific evidence has found a correlation between high levels of C-reactive protein and manic states, while more uncertain data are those concerning euthymic and depressive states (19), although some studies show the increase, decrease or absence of variations of CRP during the various phases of the disease (14). The role of C-reactive protein in the neuro-progression of the disease remains unclear.

This retrospective pilot work aimed to evaluate the relationship between the improvement of the acute symptoms of bipolar disorder and the improvement of the inflammatory marker levels C-reactive protein in hospitalized patients.

## Materials and Methods

This observational study aimed to retrospectively evaluate the relationship between the inflammatory marker CRP and bipolar disorder severity. The study was approved by the University of Siena and Area Vasta - South-East Institutional Review Board - Ethics Committee (num. ID17856; Approval Date: 05/02/2021).

Consecutive patients were enrolled according to the following criteria: (1) a diagnosis of bipolar disorder according to the Fifth Edition of the Diagnostic and Statistical Manual of Mental Disorders [DSM-5]; (2) age ≥ 18 years; (3) length of hospital stay longer than 8 days; (4) voluntary participation, ability to understand and sign consent information. Exclusion criteria were: (1) Primary diagnosis of schizophrenia, schizophrenic form disorder, schizoaffective disorder, and delusional disorder; (2) NeuRodegenerative disorders, intellectual disability, neurological diseases, history of head injury; (3) Any clinical condition that could interfere with the reliability of the assessment, such as: current infection, recent surgery, trauma, burns, neoplastic processes, joint rheumatism such as rheumatoid arthritis and rheumatic polymyalgia, autoimmune diseases such as SLE, inflammatory bowel disease, pelvic inflammatory disease, myocardial infarction, appendicitis, pancreatitis, cholangitis, pyelonephritis, gout, and tuberculosis; (4) Pharmacological treatment in progress with anti-inflammatories or corticosteroids doses.

Patients were treated with standard of care medications for bipolar disorder, mainly consisting of a mood stabilizer and/or an antipsychotic combined, when necessary, with other medications such as antidepressants, benzodiazepines, or other hypnotics.

### Endpoints

Blood tests were collected at admission to inpatient treatment (T0) and after 7 days from the admission (T1).

### Blood Tests and Psychometric Scales

CRP was collected for all patients at T0 and T1. CRP values higher than 0.5 mg/dL were considered as abnormal values. Clinical Global Impression (CGI) for Depression, Mania and Overall Bipolar Illness were registered at T0 and T1.

### Statistical Analysis

Descriptive statistical analyses are presented as mean ± standard deviation or median and interquartile range for continuous variables and frequencies and percentages for qualitative variables. Comparisons were determined through the paired *t*-test. Correlations between CRP and CGI scores were determined through Spearman's correlation coefficient at T0 and T1. Statistical significance was set at 5% (*p* < 0.05). STATA17 (StataCorp, College Station, TX, USA) was used for all statistical analyses.

## Results

Sixty-one patients (42 females and 19 males, median age 50 years, IQR: 38–63 years) were enrolled and included in the study. Thirty patients were affected by bipolar disorder type I, 31 patients by bipolar disorder type II. Other psychiatric comorbidities were present in 9 patients. Thirteen patients reported significant physical comorbidities. Complete patients' characteristics are reported in Table 1.

**Table 1:** Patients characteristics.

	**Median (IQR) or *N* (%)**
N. Patients	61
Age, years	50 (38–63)
Sex	
Female	42 (68.9%)
Male	19 (31.1%)
Diagnosis	
Bipolar disorder (type I)	30 (49.2%)
Bipolar disorder (type II)	31 (50.8%)
Other psychiatric comorbidities	9 (14.8%)
Physical comorbidities	13 (21.3%)
BMI	25.3 (22.1–27.2)
Underweight (<18.5)	4 (6.6%)
Normal (18.5–22.9)	16 (26.2%)
Overweight (23.0–24.9)	9 (14.8%)
Obese (≥25.0)	32 (52.5%)
Smoking	31 (50.8%)

Mean CRP significantly decreased during hospitalization (mean CRP at admission: 0.60 ± 0.24 mg/dL; mean CRP after 7 days: 0.42 ± 0.17 mg/ dL; *p* < 0.001; no missing values). Positive correlations emerged between CRP and CGI scores at T0 and T1 as reported in Table 2.

**Table 2:** Spearman's correlation coefficient at T0 and T1.

	**CRP T0**	**CGI OBI T0**	**CGI Depression T0**	**CGI Mania T0**
CRP T0	1.0000			
CGI OBI T0	0.7607^*^	1.0000		
CGI Depression T0	0.4017^*^	0.3871^*^	1.0000	
CGI Mania T0	0.8811^*^	0.7185^*^	0.4517^*^	1.0000
	**CRP T1**	**CGI OBI T1**	**CGI Depression T1**	**CGI Mania T1**
CRP T1	1.0000			
CGI OBI T1	0.3330^*^	1.0000		
CGI Depression T1	0.6609^*^	0.2661^*^	1.0000	
CGI Mania T1	0.4998^*^	0.4249^*^	0.4708^*^	1.0000

* *p < 0.05*.

## Discussion

We found that patients admitted to the inpatient unit reported a reduction of CRP during the first 7 days of supervised treatment, in synch with the improvement of their acute symptoms of bipolar disorder. Our results confirm those already reported in other studies in which CRP detected in the various stages of bipolar disorder disease was higher in untreated patients, possibly due to a direct protective role of medication treatment (i.e., mood stabilizers or atypical antipsychotics) against the patient's inflammatory state or due to the improvement in bipolar disease related to the use of appropriate medications (15, 16). For instance, Van den Ameele et al. found that untreated patients with BD showed disease-related inflammatory cytokine alterations, while patients in a state of euthymia and receiving a treatment with mood stabilizers such as lithium, had similar values to healthy controls, this suggesting a role of medications such as lithium in normalizing the immune system (20).

This study has several limitations. First, the observation period was short, and we were unable to report on the longer-term changes of CRP, for instance during the euthymia period. Second, the concentration of baseline CRP exhibited a large interindividual variability. Third, the sample size was small. Fourth, we were unable to establish if the decrease in CRP was specifically related to specific medications or were instead due to other factors (i.e., admission to the hospital). Finally, possible confounding effects on the CRP serum caused by minor comorbidities were not considered.

Because of the factors above, we were unable to establish if the decrease in inflammatory markers was primarily a direct effect of the medications that were prescribed during inpatient stay, or may also be related to other factors, such as the inpatient admission itself, psychoeducation, and the consequent improvement in circadian rhythms and daily routine. Indeed, the positive correlation between CRP and a higher degree of symptoms severity could be related to the activation of the stress response induced by symptoms such as increased psychomotor activity and lack of sleep, via complex interplay of endocrine, nervous, and immune mechanisms that involve activation of the sympathetic-Adreno-Medullar (SAM) axis, the Hypothalamus-Pituitary-Adrenal (HPA) axis, and the immune system (21). Nonetheless, our results confirm a relationship between CRP and clinical status, with a decrease in CRP as patients' acute symptoms improved. A recent paper evaluated the association between inflammation and specific symptoms of depression and suggested symptom-specific rather than generalized effects of inflammation on depression (22).

Studying the relationship between the improvement of the acute symptoms of bipolar disorder and the reduction of the C-reactive protein levels may hypothesize new clinically-oriented research: (1) whether and how much an early improvement of inflammatory markers indicates the efficacy of a new treatment; (2) whether and which anti-inflammatory treatments can reduce bipolar disorder symptoms; and (3) if the decrease of inflammatory markers is directly correlated with an improvement of bipolar disorder symptoms or if the improvement of inflammatory markers indicates the change of a third factor (e.g., an infection, a change in microbiota, etc.) that is correlated with the symptoms of bipolar disorder and that may become a future target of bipolar disorder treatment.

Specifically, a pooled analysis of population-based cohort studies, reported that higher concentrations of CRP were strongly associated with the presence of four physical symptoms (loss of energy, sleep problems, changes in appetite, fatigue) and one cognitive symptom (lack of interest in doing things). Following these suggestions, future studies could further investigate the relationship between inflammatory markers, such as CRP, and specific symptoms of bipolar disease.

## Conclusions

A significant decrease in CRP was observed in patients admitted to the inpatient unit during the first 7 days of treatment, as their symptoms of bipolar disorder improved. It remains to be established which specific factor contribute to the decrease in CRP and whether the decrease of CPR is related to an improvement of bipolar disorder symptoms or vice-versa.

## Data Availability Statement

The original contributions presented in the study are included in the article/supplementary materials, further inquiries can be directed to the corresponding author/s.

## Ethics Statement

The studies involving human participants were reviewed and approved by the University of Siena and Area Vasta - South-East Institutional Review Board - Ethics Committee. The patients/participants provided their written informed consent to participate in this study.

## Author Contributions

AC, DK, and AF contributed to conception and design of the study. AS organized the database. GB and AS performed the statistical analysis. AC, DK, and SB wrote the first draft of the manuscript. AG and GB wrote sections of the manuscript. All authors contributed to manuscript revision, read, and approved the submitted version.

## Conflict of Interest

AF is/has been a consultant and/or a speaker and/or has received research grants from Angelini, Apsen, Boheringer Ingelheim, Daiichi Sankyo, Doc Generici, Glaxo Smith Kline, Italfarmaco, Lundbeck, Janssen, Mylan, Neuraxpharm, Otsuka, Pfizer, Recordati, Sanofi Aventis, Sunovion, Vifor. AC is/has been a consultant and/or a speaker for Angelini, Glaxo Smith Kline, Lundbeck, Janssen, Otsuka, Pfizer, Recordati. The remaining authors declare that the research was conducted in the absence of any commercial or financial relationships that could be construed as a potential conflict of interest.

## Publisher's Note

All claims expressed in this article are solely those of the authors and do not necessarily represent those of their affiliated organizations, or those of the publisher, the editors and the reviewers. Any product that may be evaluated in this article, or claim that may be made by its manufacturer, is not guaranteed or endorsed by the publisher.
